# The Virtual Brain: Modeling Biological Correlates of Recovery after Chronic Stroke

**DOI:** 10.3389/fneur.2015.00228

**Published:** 2015-11-02

**Authors:** Maria Inez Falcon, Jeffrey D. Riley, Viktor Jirsa, Anthony R. McIntosh, Ahmed D. Shereen, E. Elinor Chen, Ana Solodkin

**Affiliations:** ^1^Department of Anatomy and Neurobiology, University of California Irvine School of Medicine, Irvine, CA, USA; ^2^Department of Neurology, University of California Irvine School of Medicine, Irvine, CA, USA; ^3^Institut de Neurosciences des Systèmes, Faculté de Médecine, Aix-Marseille Université, Marseille, France; ^4^INSERM UMR1106, Aix-Marseille Université, Marseille, France; ^5^Rotman Research Institute, Baycrest Health Sciences, University of Toronto, Toronto, ON, Canada

**Keywords:** stroke, brain dynamics, graph theory, computational biophysical modeling, connectome, brain networks, imaging, MRI

## Abstract

There currently remains considerable variability in stroke survivor recovery. To address this, developing individualized treatment has become an important goal in stroke treatment. As a first step, it is necessary to determine brain dynamics associated with stroke and recovery. While recent methods have made strides in this direction, we still lack physiological biomarkers. The Virtual Brain (TVB) is a novel application for modeling brain dynamics that simulates an individual’s brain activity by integrating their own neuroimaging data with local biophysical models. Here, we give a detailed description of the TVB modeling process and explore model parameters associated with stroke. In order to establish a parallel between this new type of modeling and those currently in use, in this work we establish an association between a specific TVB parameter (long-range coupling) that increases after stroke with metrics derived from graph analysis. We used TVB to simulate the individual BOLD signals for 20 patients with stroke and 10 healthy controls. We performed graph analysis on their structural connectivity matrices calculating degree centrality, betweenness centrality, and global efficiency. Linear regression analysis demonstrated that long-range coupling is negatively correlated with global efficiency (*P* = 0.038), but is not correlated with degree centrality or betweenness centrality. Our results suggest that the larger influence of local dynamics seen through the long-range coupling parameter is closely associated with a decreased efficiency of the system. We thus propose that the increase in the long-range parameter in TVB (indicating a bias toward local over global dynamics) is deleterious because it reduces communication as suggested by the decrease in efficiency. The new model platform TVB hence provides a novel perspective to understanding biophysical parameters responsible for global brain dynamics after stroke, allowing the design of focused therapeutic interventions.

## Introduction

Heterogeneity of functional outcomes following stroke remains a major limitation to stroke rehabilitation. While the majority of stroke survivors suffer from motor impairment, particularly in the upper extremities (1), the degree and type of this impairment and the level of recovery following rehabilitation are highly variable (2). The functional basis for variation in patient deficits is still poorly understood, and there is no consensus on a theoretical or empirical framework for linking brain injury to functional deficits (3). In order to address this issue, recent approaches in stroke rehabilitation have aimed at the development and the optimization of individualized treatments that maximize long-term functional gains (4, 5).

To this end, different theoretical approaches have been used. The most general method has probed stratification measures based on patient demographics, behavioral outcomes, affective states, brain function, and lesion characteristics (4–6). None have been shown as a reliable biomarker. Particularly noticeably has been the presence of an inconsistent relationship between brain lesion and the resulting functional deficits (6), likely due to the inherent complexity of damage in a highly interconnected brain.

Researchers have thus turned to network analysis to understand stroke (7–9). In this approach, one of the goals is to explain the observed variations after stroke and predict recovery. Interestingly, the initial efforts with network analysis focused on alterations to specific pathways as the key links to understand behavior (8, 10). For example, while some functional connectivity studies showed that lesions within the motor areas can cause dysfunction of remote brain regions (11–13), others showed a relationship between improved motor function and strengthening interhemispheric and intrahemispheric connectivity involving the primary motor cortex (14). An important issue in interpreting such relationships is that the changes may reflect either the abnormal functioning of a damaged network or the formation of a different network that results in new behavioral patterns.

Furthermore, while these initial studies have been an important development, their main limitation is that they assume stable, localized changes within specific sub-networks, obliterating global changes, with the consequence that these potential biomarkers have been very adequate as descriptors at the group level but not in individual patients (15).

Recently, the neuroimaging community has begun to focus on connectomics, or the mapping of all connections at the whole-brain level. These connectomes, derived from structural [diffusion tensor imaging (DTI)] or functional outputs (fMRI and EEG), have recently been termed “big data,” referring to datasets that require the generation of large amounts of multimodal imaging data, (including raw, preprocessed, and intermediate data), for a high number of subjects (16). These initiatives span normal function [Human Connectome Project (17), CONNECT (18), Brainnetome (19), development [National Institutes of Health (NIH) Pediatric Database] and brain disorders such as Alzheimer’s disease (Alzheimer’s Disease Neuroimaging Initiative)].

In order to help interpret such large datasets, graph theory is increasingly used to distinguish inherent patterns that likely correlate with brain networks at the whole-brain level. Using connectomics and graph theory, specific brain regions can be understood as nodes (20), and lesions can be understood as damage to nodes and/or the connections among them. With these methods, stroke has been shown to produce changes in both structural and functional network connectivity, particularly related to the organization of “hubs,” or highly interconnected nodes (21, 22). Graph theory provides an assessment of the changes at an organizational level. However, this approach still suffers from some limitations, mainly the inability to determine dynamical changes in a constantly changing brain and the lack of concrete biophysical substrates for understanding those dynamics. Consequently, according to Smith et al., one of the major challenges in the field of functional connectomics “will be to enable application of biologically interpretable models using large numbers of nodes in a robust and practical way” (9).

In other words, although tackling questions about brain network dynamics in both healthy and stroke populations requires a great deal of data, simply collecting more data is not itself an answer. While these efforts provide the necessary empirical foundation, they lack a computational and theoretical framework with quantitative tools to link these multiple datasets to “reconstruct” the brain and provide the link between these data and the brain function of individuals.

In this context, novel theoretical perspectives have been proposed based upon the nature of the brain as a large-scale network (3, 23–25). The implementation of the framework has been significantly accelerated by The Virtual Brain (TVB), a novel large-scale neural modeling platform (26–28). TVB uses neuroimaging data to parameterize a model and because individual data is used, the individual person’s brain can become a “virtual brain.”

The Virtual Brain (thevirtualbrain.org) was developed as a platform for modeling the dynamics of large-scale neural systems (3, 29). TVB integrates structural long-range connectivity generated from empirical DTI data with mesoscopic, or local level models [at each node or region of interest (ROI)]. By combining these two scales (global connectivity with local dynamics), TVB is able to predict and simulate an individual’s brain activity, essentially modeling a virtual representation of their brain. TVB thus lies at the intersection of experimental and theoretical neurosciences, making it well positioned to provide a link between population and individual datasets.

The models available in TVB integrate the anatomical connectivity between parts of the brain (provided by DTI) and the dynamics of local neural populations (embedded in the platform). Using these models, TVB has the flexibility to generate simulated data ranging from local field potentials to EEG and fMRI BOLD signals, allowing for a multimodal link between simulated and empirical data. The scalable architecture of TVB allows us to include neurophysiological information (e.g., receptor distributions and ion channels) adding another level of detail and bringing the model’s behavior closer to the real brain. Spatiotemporal motifs as present in empirical EEG/fMRI data can be reproduced to a large degree (29, 30). Because biophysical parameters are invisible to brain-imaging devices, TVB acts as a “computational microscope” that allows the inference of internal states and processes of the large-scale model.

The Virtual Brain therefore serves as a powerful research tool that has the potential to utilize big data and to develop and test advanced theories of brain dynamics. The individualization of TVB allows the creation of one model per person and systematically assesses the modeled biophysical parameters related to individual differences. The natural extension of this approach goes further into clinical applications, deriving parameters that both relate to biophysics and predict clinical outcome, making TVB an ideal tool for addressing limitations in stroke research.

The objective of this manuscript is twofold:

(1) To give a thorough overview of the modeling method employed using TVB as it pertains to stroke, with the goal of providing details for those interested in using it in the context of stroke.
(2) To provide a link between one of the TVB parameters (long-range coupling) to current whole-brain analytical approaches based on graph analysis.

## Materials and Methods

### Subjects

Twenty individuals with ischemic stroke in the middle cerebral artery territory (41.13 ± 23.78 months postonset) and 10 age-matched controls were recruited for the study. Demographics for all stroke subjects are shown in Table 1.

**Table 1:** **Demographics and stroke characteristics of the stroke cohort**.

Subject	Age	Sex	Handedness	Affected hemisphere	Affected hand	Stroke location	Stroke volume (mm^3^)
1	41	F	Right	Right	ND	Cort	22,495.0
2	54	F	Right	Left	D	Cort/subcort	49,078.0
3	57	M	Right	Left	D	Cort/subcort	17,411.0
4	57	M	Right	Left	D	Cort/subcort	38,703.0
5	54	F	Right	Left	D	Subcort	27,677.0
6	50	M	Right	Right	ND	Subcort	3,570.0
7	23	M	Right	Left	D	Subcort	560.0
8	55	F	Right	Right	ND	Cort	6,781.0
9	68	M	Right	Left	D	Subcort	1,988.3
10	56	F	Right	Left	D	Subcort	6,239.7
11	46	M	Right	Left	D	Subcort	325.0
12	56	F	Left	Right	D	Cort/subcort	60,669.0
13	37	M	Right	Left	D	Cort/subcort	83,406.2
14	62	M	Right	Left	D	Subcort	22,154.8
15	57	M	Right	Right	ND	Cort/subcort	25,392.0
16	66	M	Right	Left	ND	Cort/subcort	19,927.0
17	61	M	Right	Left	D	Subcort	978.0
18	74	M	Right	Left	D	Cort/subcort	63,642.0
19	67	F	Right	Right	ND	Subcort	588.0
20	74	F	Right	Left	D	Cort/subcort	44,892.0

*D, dominant hemisphere; ND, non-dominant; Cort, cortical; subcort, subcortical*.

### Imaging Acquisitions

Magnetic resonance images were collected using a 3-T Philips scanner and an eight-channel SENSE head coil for signal reception and body coil transmitter for signal excitation. The following sequences were used:

1. High-resolution anatomical images (T1-w): three-dimensional (3D) Magnetization Prepared Rapid Gradient Echo sequence, FOV = 250 × 250, resolution = 1 mm × 1 mm × 1 mm, SENSE reduction factor = 1.5, TR/TE = 7.4/3.4 ms, flip angle = 8, sagittal orientation, and number of slices = 301 covering the whole brain.
2. Diffusion Tensor Imaging (DTI): FOV = 224 × 224, TR/TE = 13,030/55, 72 slices, slice thickness = 2 mm, resolution = 0.875 × 0.875 × 2, *b* = 1,000 s/mm^2^ (and *b* = 0), 32 diffusion directions.
3. Functional imaging acquisition at rest (rsfMRI): whole brain (37 slices), single-shot echo-planar MR (EPI), slice thickness = 4.0 mm, FOV = 230 × 230, voxel size = 2.8 mm × 2.8 mm, TR/TE = 2,000/20 ms, and duration = 5 min.

### Resting State fMRI Preprocessing

Resting state fMRI (rsfMRI) preprocessing analysis was performed using AFNI functions (31) and included the following steps:

1. Motion correction using a six-parameter 3D registration of functional and anatomical data sets (32).
2. Three-dimensional spatial registration to a reference acquisition from the first fMRI run.
3. Registration of functional images to the anatomical volume.
4. Despiking of the time series.
5. Mean normalization of the time series.
6. Inspection and censoring of time points occurring during excessive motion (>1 mm) (33).
7. Regression of cerebrospinal fluid and white matter signals to remove slow drifts in the fMRI signal.

### Preprocessing: Structural Connectivity

#### Brain Parcelation

**Figure 1:**
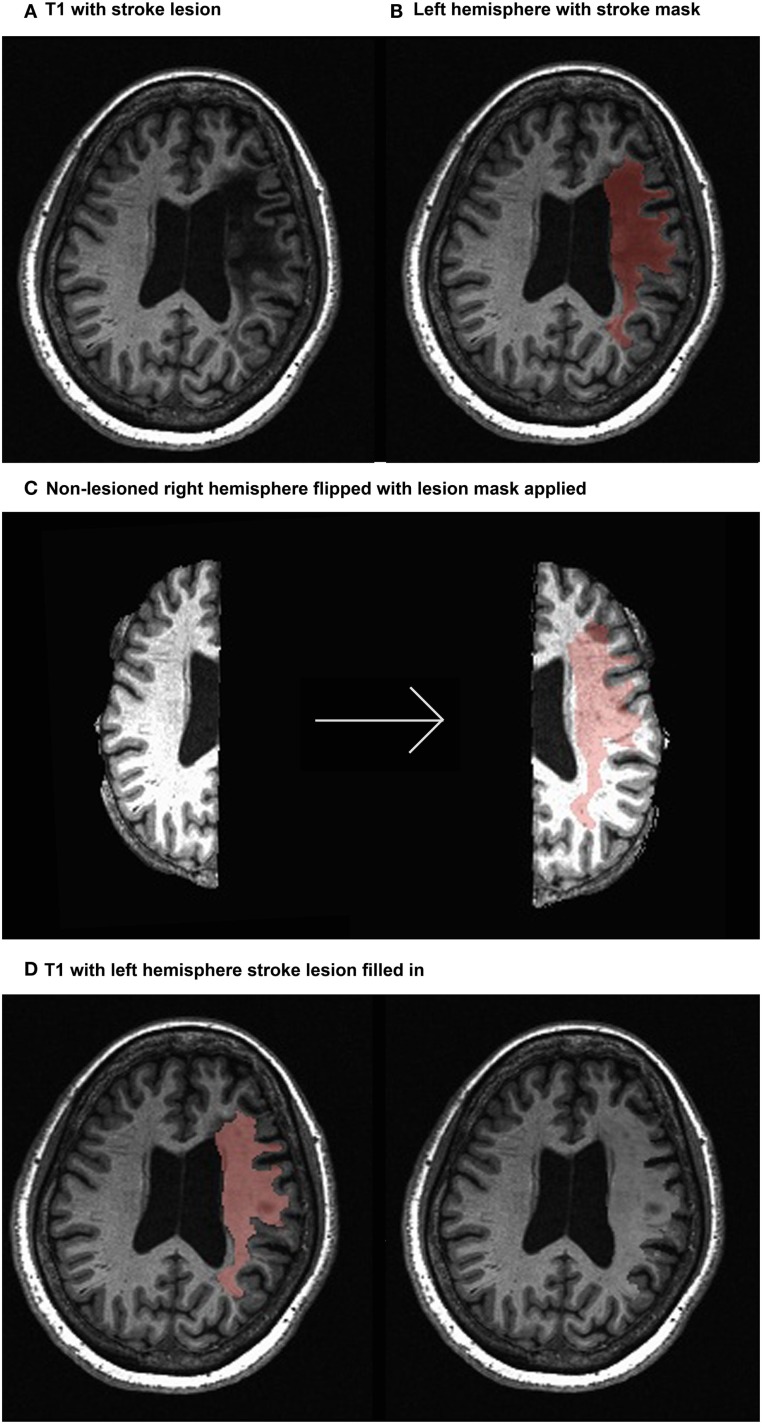
**Virtual brain transplant method**. Virtual brain transplant is done in stroke cases with cortical damage with the goal of being able to parcellate the brain. This graphic representation summarizes the process of replacing the damaged portion of the brain with the homologous non-stroke tissue. **(A)** T1-w image showing the lesion (left hemisphere) of one subject. **(B)** Close-up of the left hemisphere, demarcating the lesion mask in red. **(C)** Segregation of the right and left hemispheres (left) and after the right hemisphere has been flipped having the lesion mask applied (right). **(D)** Depiction of the tissue from the right hemisphere applied to the lesion in the left hemisphere (left) and the resulting transplanted brain volume (right).

Parcellating image data that contain lesions with the use of semiautomated schemes produce inaccurate results due to the absence of tissue and consequent mechanical deformation. We therefore developed The Virtual Brain transplant (VBT). This method effectively replaces the lesion produced by the cortical stroke with T1-w images of brain tissue from the contralesional hemisphere from the same subject (34). This method allows us to use a semiautomated parcelation scheme subsequent to the transplant. The VBT process consisted of the following steps (Figure 1):

1. Lesion segmentation by hand.
2. The high-resolution anatomical T1-w brain images and lesion masks were uploaded to a transplantation pipeline, which dissected the MRI brain tissue from the non-lesioned hemisphere homologous to the lesion, and transplanted it into the lesioned hemisphere at the site of the lesion, filling in the missing portions of the brain.
3. After the initial transplant was done, manual corrections in the interface between the native and transplanted T1-w images were performed.
4. The brain was segmented into 83 cortical and subcortical regions using the Lausanne 2008 (Freesurfer) parcelation scheme within the Connectome Mapper Toolkit (35, 36).

#### T1-w to DTI Alignment

The T1-w anatomical image was then aligned to a reference *b* = 0 s/mm^2^ DTI image, using a six degrees of freedom linear transformation with FSL’s FLIRT function (37). This transformation was also applied to the Freesurfer parcellations.

#### DTI Tractography

We performed the following steps:

1. DWI was aligned to the same reference *b* = 0 s/mm^2^ image used to align the corrected T1-w via VBT to DTI. Distortions caused by eddy currents and head motion were corrected using the FSL eddy current correction (12 degrees of freedom linear transformation), and the diffusion gradient vectors rotated accordingly (38). That is, the T1-w images with the “transplanted masks” are used to supply the region of interest landmarks for tractography but do not directly impact the tractography algorithm as the transplant is not performed in the DWI space.
2. The diffusion-weighted images were resampled to 2mm isotropic resolution (39).
3. White matter deterministic tractography of DTI data was performed in Trackvis software (39) using the FACT algorithm (40). Threshold values of a maximum of 60° turning angle and a minimum of 0.20 fractional anisotropy (FA) were used as stopping criteria for the tracking algorithm. These thresholds take into account the decrease in signal in regions with the lesion. The FA threshold is particularly useful in terminating tracks before they enter regions containing the lesion. These regions, filled with CSF, have FA values close to zero. Therefore white matter pathways ordinarily connecting two ROIs will not be tracked if the ROI is completely lesioned, despite appearing intact in the transplanted T1-w image from which the parcelation is made. If a parcelation is partially compromised by the lesion then white matter pathways will also be partially tracked as reflected by a lesser number of streamlines.

#### Generation of Structural Connectivity Matrices

Using the Connectome Mapper Toolkit, two connectivity metrics were extracted for each pathway in order to generate two structural connectivity matrices that quantify connectivity between all pairs of the cortical regions for each subject:

1. Weights, defined as FA × number of streamlines in the pathway (note that per the white matter deterministic tractography of DTI data, pathways connecting regions impacted by the lesion will show a decreased number of streamlines and potentially altered FA). This metric reflects the maximum rate of transmission of information through edges (41). The number of streamlines in the pathway was assessed using the deterministic FACT algorithm.
2. Lengths of the individual tracts, defined in millimeters, were derived after smoothing the tractography with a B-Spline filter (39).

These matrices are symmetrical, as connections using DTI are considered unidirectional (30).

### Modeling with TVB

Modeling with TVB involves three initial steps, namely the import of individual structural connectivity matrices (obtained as described earlier), the selection of a biophysical local model, and the choice of relevant biophysical parameter values. TVB has several types of local models available, each one taking into account different biophysical parameters. Hence, whereas some are focused on field potentials [Stefanescu–Jirsa two dimensional (2D) and Stefanescu–Jirsa 3D (SJ3D)], others are focused on firing rates (Wilson–Cowan, Brunel–Wang, and Jansen–Rit) or are phenomenological (Generic 2D, Kuramoto, and Epileptor). In our previous efforts, since we simulated the BOLD response, the mesoscopic model used was the SJ3D, one of the more complex and refined models in the repertoire of TVB.

The reasoning behind this choice was not only the obvious relationship between the BOLD response and local field potentials (42–44) but the additional fact that the BOLD signal has poor time resolution and the model does not rely heavily on synaptic delays. Concretely, the SJ3D model is a reduced form of the Hindmarsh–Rose model (43), which forecasts individual neuronal behavior. The SJ3D model predicts local dynamics using six differential equations that include variables representing *physiological properties* such as neuron membrane potentials, transport of ions across the membrane through fast and slow ion channels, and the dynamic coupling of excitatory and inhibitory neuronal populations.

**Figure 2:**
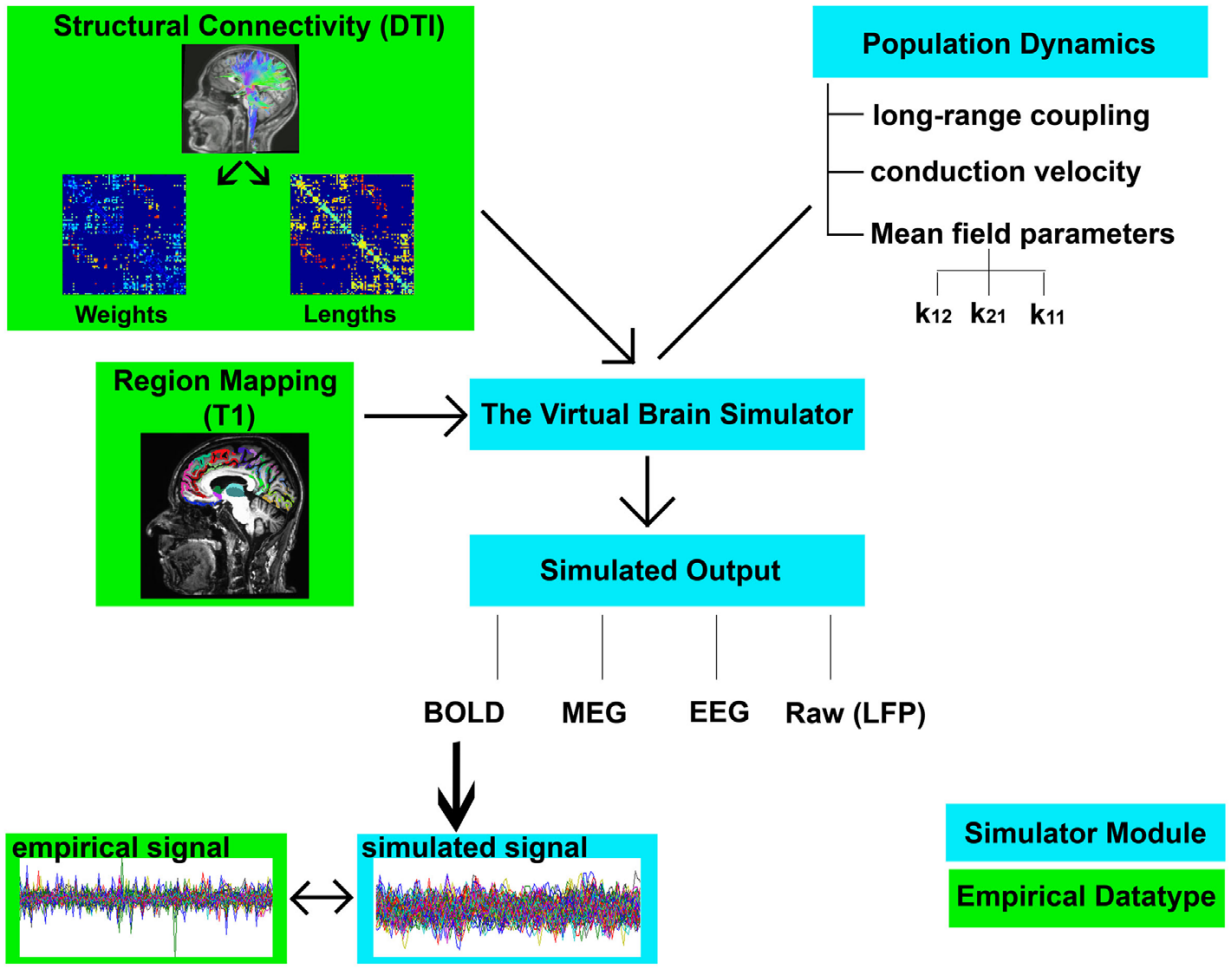
**Flowchart of TVB modeling**. Graphic representation depicting the elements involved in TVB modeling. Components shown in green boxes represent empirically collected data. Elements shown in blue boxes represent modeling components within the TVB platform. Empirical input to the TVB consists of two structural connectivity matrices (weights and lengths) derived from DTI and a brain parcelation derived from T1-w acquisition. Modeling within TVB includes both global and local parameters resulting in the simulation of biological signals including BOLD. Finally, the reliability of the simulation is then compared to the empirical signals.

**Figure 3:**
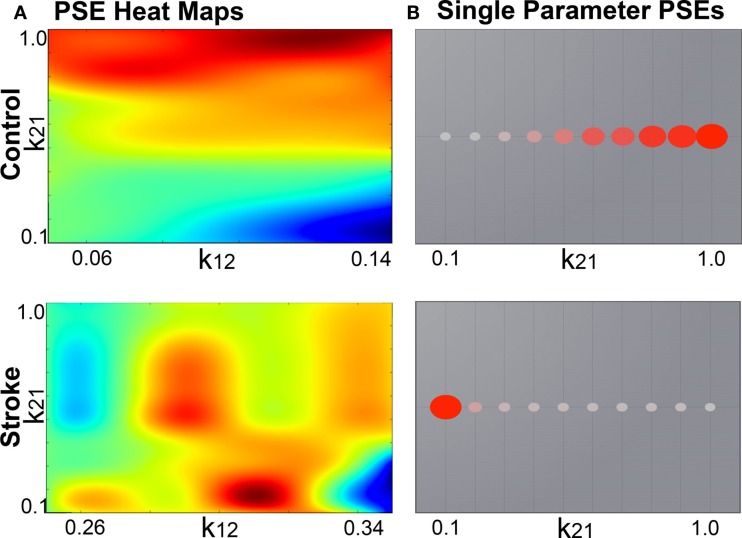
**Example global parameter space explorations in healthy and stroke cases**. This figure represents the two viewing options for multiple parameter or single parameter explorations. **(A)** Parameter explorations of the *K*_12_ and *K*_21_ variables (coupling between inhibitory and excitatory populations) in one healthy control (top) and one stroke case (bottom). Heat maps depict the distribution of system variance, with hotter colors indicating values of parameters that yield higher variance. High resolution of heat maps allows for identification of precise parameter values related to high variance. **(B)** Parameter exploration of the *K*_21_ variable alone, after optimization has been completed. Colored circles depict degree of variance at each value of *K*_21_.

**Figure 4:**
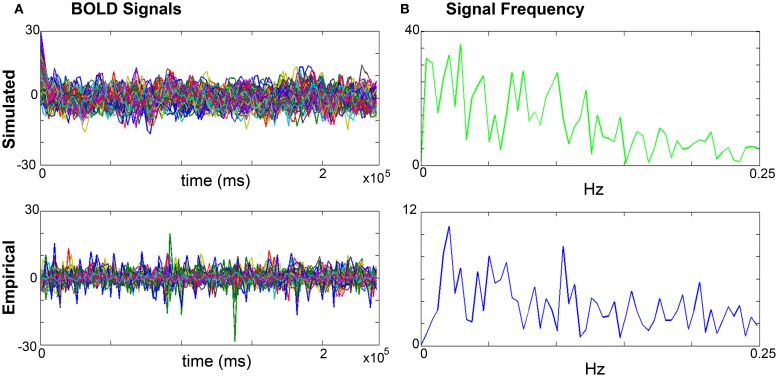
**Comparisons of simulated and empirical signals: amplitude and frequency**. **(A)** BOLD time series: example of a simulated (top) and empirical (bottom) time series. Note the similarity of amplitudes as indicated by the maxima and minima. **(B)** Frequency: example frequency distribution graphs for primary motor cortex (M1) of the simulated (top) and empirical (bottom) time series where both signals have similar profiles and peaks.

**Figure 5:**
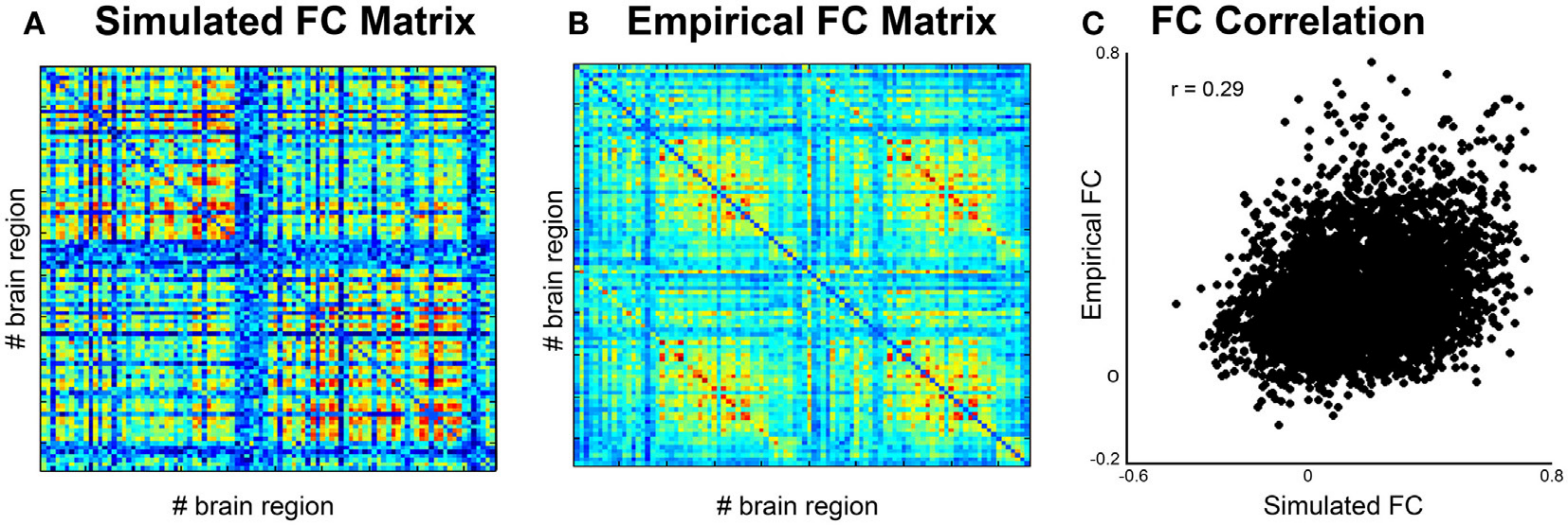
**Comparison of simulated and empirical signals: phase**. **(A)** Functional connectivity matrix from simulated data modeled from one subject. **(B)** Average functional connectivity matrix from empirical data from all healthy subjects. **(C)** Correlation of functional connectivity between simulated (*x*-axis) and empirical (*y*-axis) time series.

The sequential steps for modeling in TVB are as follows (graphical depiction can be found in Figure 2):

1. Importing the two metrics derived from individualized SC matrices [weights (FA × number of fibers) and lengths] representing connections between regions, along with the T1-w structural data providing individual brain topology.
2. Parameter space exploration: the goal of this process is the optimization of the model parameters. When applying TVB methodology to stroke, one can classify the numerous parameters included in the modeling into two categories: global parameters that will model brain dynamics between nodes, and local parameters that will describe brain dynamics within nodes. In the first category, the two main parameters to optimize are conduction velocity and long-range coupling. Likewise the biophysical parameters within the SJ3D model to be used are those providing the coupling between excitatory and inhibitory populations within the local regions: *K*_11_ (excitatory on excitatory), *K*_12_ (excitatory on inhibitory), and *K*_21_ (inhibitory on excitatory). This exploration systematically explores the entire range of available values for each parameter and identifies the value with the highest overall distribution of variance (Figure 3) as the optimal parameter value to be used on each individual for the actual signal simulation. The order of optimization can be done as follows:
  - Long-range coupling and conduction velocity: starting ranges are 0.001–0.1 global coupling and 1–100 conduction velocity.
  - *K*_12_ and *K*_21_: starting ranges are 0–1.0 for both. *K*_12_ is optimized first, and the identified value is then used when optimizing *K*_21_.
  - *K*_11_: starting range is 0–1.0.
3. Simulating the BOLD response: based on the values obtained in the parameter exploration, simulation of the BOLD time series should reflect the same duration (4 min) and sampling rate (TR = 2 s) of the empirical MRI acquisition. Noise is added to each node. The noise to be used is white with Gaussian amplitude (mean = 0, standard deviation = 1). Numerical integration of the system is performed using stochastic Heun’s method (45), with an integration step size of 0.0122 ms.
4. Validating the simulated brain signals: this is done by comparing the simulated and empirical time series in terms of their amplitude, frequency, and phase.
  - Amplitude: the range is calculated by identifying the highest and lowest peaks present in the time series across all regions. The overall mean is calculated by averaging the mean amplitude per region across all regions. Mean amplitudes should be similar. An example is shown in Figure 4A.
  - Frequency is computed via fast Fourier transforms of the time series with Matlab’s “fft” function with an fs of 0.5 Hz to determine the range, profile, and peak frequencies. The maximum frequency for simulated signals should be around 0.25 Hz that coincides with the empirical BOLD responses. An example is shown in Figure 4B.
  - Phase can be done by calculating the pair-wise covariance of the time series for each region for each subject (30) using the “corr” function in Matlab, which results in a functional connectivity matrix for each subject. In order to smooth the data, one can average all matrices from groups of interest to obtain a group control matrix and then calculate the pair-wise linear correlation coefficient between the simulated functional connectivity matrix for each individual to the group (Figure 5). Results from this analysis should reveal similar phases between empirical and simulated signals. Significance of the correlation can be achieved via Fisher *Z*-transformation.

### Comparison Between Healthy Controls and Stroke

We found an increase in long-range coupling in the stroke group compared to healthy controls. The meaning of long-range coupling is not intuitive, especially when compared to other parameters more closely linked to biophysical features, such as conduction velocity, channel dynamics, and the coupling between excitatory and inhibitory neuronal populations. The long-range coupling function is applied to the activity propagated between brain region regions by the structural pathways before it enters the local dynamic equations of the model. Its primary purpose is to rescale the incoming activity to a level appropriate to model. At a more intuitive level this parameter describes the balance between the global and the local dynamics. In other words, an increase in long-range coupling suggests a preponderance of local over long-range brain dynamics.

In order to put this parameter in the context of current network analytical approaches, in this study we determined the relationship between the modeled long-range coupling in stroke cases with structural network metrics derived from graph analysis including degree centrality, betweenness centrality, and global efficiency.

### Graph Analysis

#### Graph Analysis Metrics

Based on the deterministic tractography performed for each individual subject, a binary adjacency matrix *A_ij_* was generated whose elements represent the connections (edges) between nodes *i* and *j* (46–48). From these matrices, three measures of functional integration were obtained: average degree centrality, average betweenness centrality, and global efficiency as others have done (49–51), using the NetworkX software (52) [mathematical notation adapted from (20)]:

1. Average degree centrality is the number of nodes adjacent to node *i*, averaged across all nodes in the graph (53):
  $$k_{\mathrm{av}}=\frac{1}{n}\sum_{i\in N}k_{i}=\frac{1}{n}\sum_{i,j\in N}a_{\mathrm{ij}}$$
  where *n* is the number of nodes in the graph, and *N* is the set of those nodes; *k_i_* is the degree centrality for node *i*, and *a_ij_* equals 1 when nodes *i* and *j* are the nearest neighbors and zero otherwise. This is the simplest measure of centrality and is commonly used to discriminate between well-connected nodes (hubs) and less well-connected nodes (51).
2. Average betweenness centrality refers to the fraction of shortest paths between any pair of nodes in the network that travel through a given node averaged across all nodes (54):
  $$b_{\mathrm{av}}=\frac{1}{n}\sum_{i\in N}b_{i}=\frac{1}{n}\sum_{i\in N}\frac{2}{\left(n-1)(n-2\right)}\sum_{\begin{array}{c} h,j\in N\\ h\neq j,h\neq i,j\neq i \end{array}}\frac{p_{\mathrm{hj}}(i)}{p_{\mathrm{hj}}}$$
  where *b_i_* is the betweenness centrality for node *i*; *p_hj_* is the number of shortest paths between nodes *h* and *j*, and *p_hj_*(*i*) is the number of shortest paths between *h* and *j* that pass through node *i*. This is the oldest and most commonly used measure of centrality (51) where “shortest” refers to the path between two nodes that contains the least number of intermediate nodes.
3. Global efficiency is the average of the inverse of the shortest path length between all nodes (minimum number of edges traversed to connect one node to another) (21, 53):
  $$E=\frac{1}{n}\sum_{i\in N}E_{i}=\frac{1}{n}\sum_{i\in N}\frac{\sum_{i\in N,j\neq i}d_{\mathrm{ij}}^{-1}}{n-1}$$

where $d_{\mathrm{ij}}^{-1}$ is the inverse of the shortest path length between nodes *i* and *j*. For binary matrices, a network where each node has a direct connection to all other nodes in the graph has maximal global efficiency, equal to 1, while a partially disconnected network has lower global efficiency (49).

#### Comparison of Graph Analysis Metrics Between Groups

To test for differences in degree centrality, betweenness centrality, and global efficiency between healthy and stroke cases, we used the Wilcoxon-rank sum test. Significance threshold was set to *P* = 0.017 (Bonferroni correction). A simple linear regression analysis was used to correlate TVB long-range coupling (independent variable) with graph analysis metrics (dependent variables).

## Results

### Comparison of Graph Analysis Metrics Between Stroke Cases and Healthy Controls

**Figure 6:**
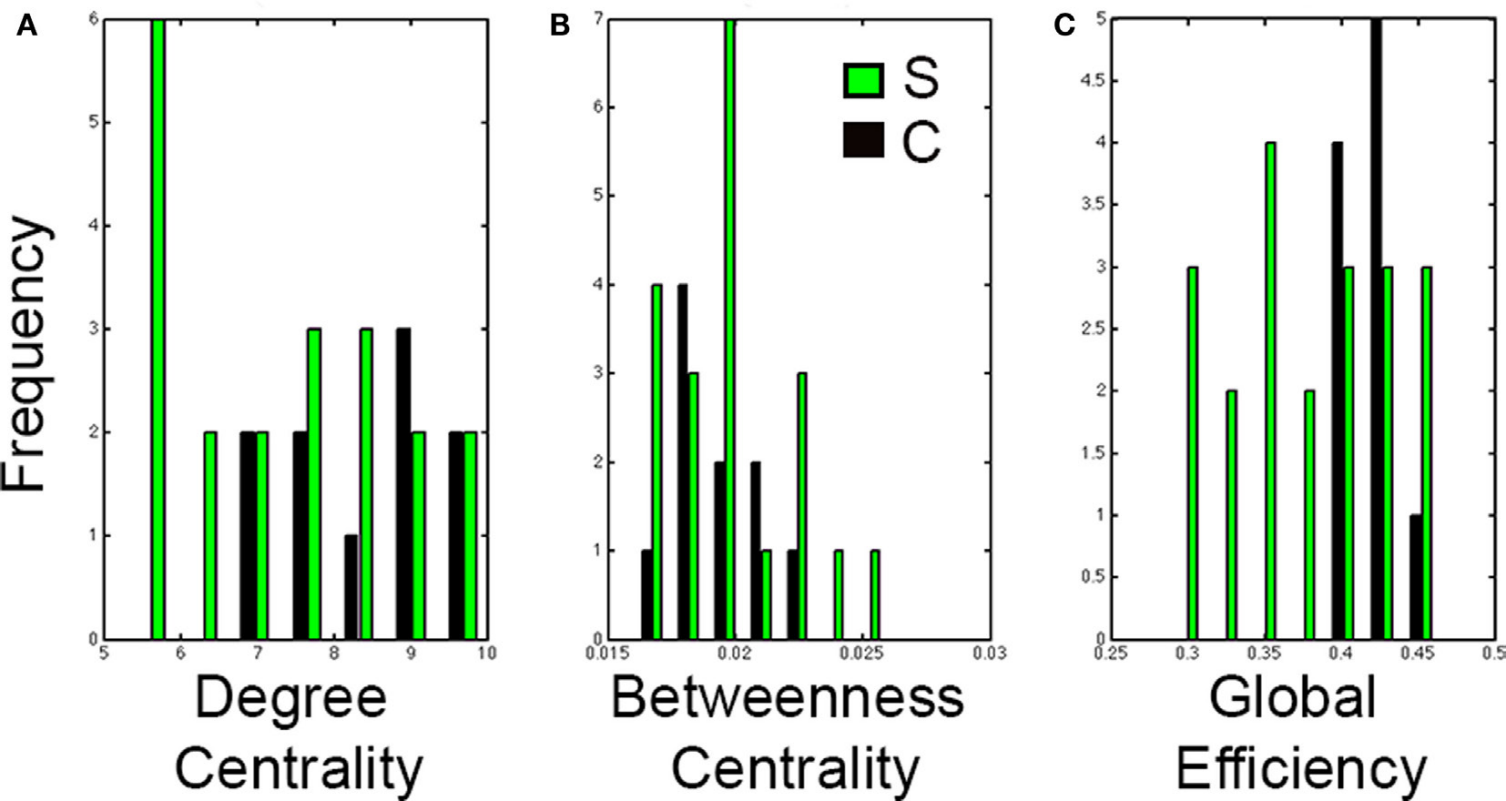
**Distributions of graph analysis metrics in control and stroke cases**. Distribution graphs comparing the control (black) and stroke (green) cases for **(A)** Degree centrality, **(B)** Betweenness centrality, and **(C)** Global efficiency. Note that distributions in stroke shift to the left for global efficiency but not for degree centrality nor for betweenness centrality.

Results from the Wilcoxon-rank sum test showed no significant differences between healthy controls and stroke cases in degree centrality (*P* = 0.11), betweenness centrality (*P* = 0.86), or global efficiency (*P* = 0.0822). However, the distributions of each graph analysis metric between the two groups showed differences (Figure 6). Specifically, global efficiency showed a trend toward lower values in stroke cases compared to controls (*P* = 0.04) but not degree centrality (*P* = 0.22) nor betweeness centrality (*P* = 0.95). While there was not a statistical difference in distribution of degree centrality between healthy and stroke populations, a large amount of subjects showed lower values of degree centrality.

### Correlation Between Long-Range Coupling and Graph Analysis Metrics

**Figure 7:**
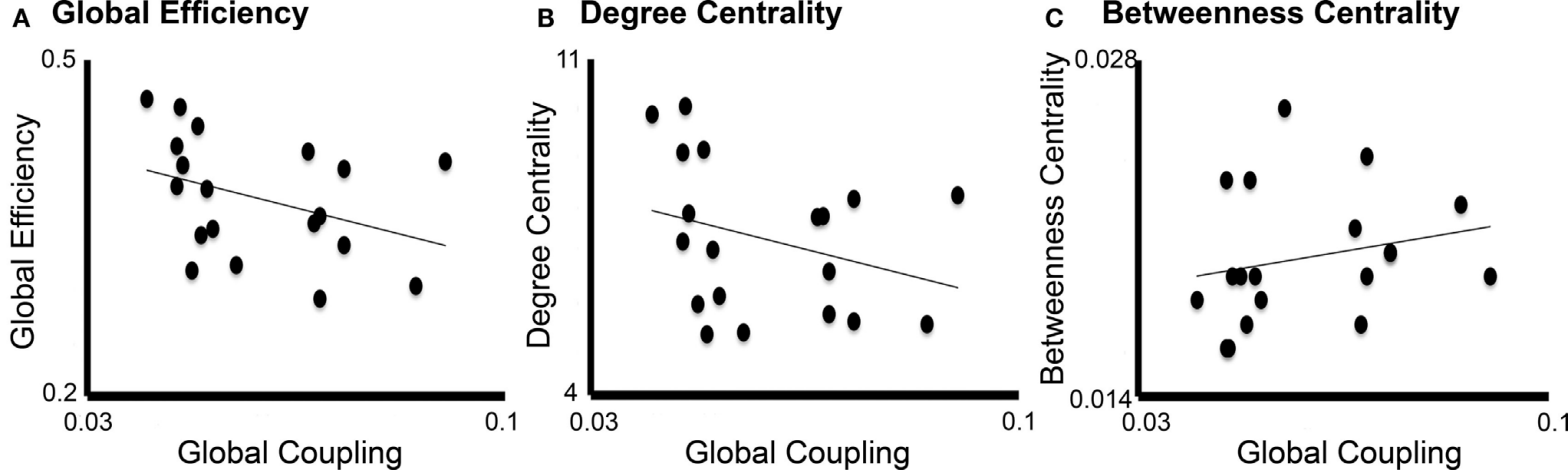
**Correlation between global coupling and graph analysis metrics**. Correlation graphs between global coupling (*x*-axes) and graph analysis metrics (*y*-axes): **(A)** Global efficiency, **(B)** Degree centrality, and **(C)** Betweenness centrality. Only global efficiency correlated significantly with long-range coupling (*P* = 0.038) but not degree centrality (*P* = 0.7) or betweenness centrality (*P* = 0.6).

Linear regression analysis showed that the only graph analysis metric associated with the TVB long-range coupling parameter was global efficiency (Figure 7). That is, higher values of global coupling were correlated with lower values of global efficiency (*t* = −2.19, *P* = 0.038). There was no significant correlation between global coupling and degree centrality (*P* = 0.7) or betweenness centrality (*P* = 0.6).

## Discussion

We have demonstrated that TVB can be a novel tool for identifying biophysical biomarkers of stroke recovery, showing that (1) the parameters associated with TVB modeling directly link structural imaging data to biophysical processes associated with brain dynamics; (2) the models are individualized, as they are based on the specific structural connectome from each person; and (3) TVB parameters can be correlated with other metrics not currently associated with biological parameters (i.e., graph analysis metrics). Importantly, this study harnessed the relationship between TVB and graph analysis, wherein the latter supplies an additional description of changes in relationships between different brain regions, while TVB supplies the neurobiological mechanisms responsible for them. The outlined steps using TVB offer a unique method, providing a new dimension to the study of stroke.

### TVB Integrates Macroscopic and Mesoscopic Levels to Predict Brain Dynamics

There is currently no way to directly measure the local parameters modeled in TVB in humans, whereas global measures derived from imaging data have been used as potential biomarkers of stroke recovery (6, 55), the parameters considered within TVB at the local level represent a dimension reduction derived from processes at the cellular or even molecular levels. That is, the mesocopic level represents the transitional state between the macro- and microscales (56). Thus, these parameters better inform us of underlying brain mechanism responsible for brain dynamics that current imaging analyses are unable to access, such as dynamics between excitatory and inhibitory neuronal populations and ion channel properties. In this way, TVB can assist to generate hypotheses associated with basic mechanisms that are responsible for the changes in brain dynamics associated with stroke.

In this context, it is important to mention that TVB can have wide applicability in the clinical setting because the input required for its operation can be minimal. In ideal circumstances, the experimental data needed are T1-w, fMRI (EEG or MEG), and DTI. However, some of these categories may not be necessary when only physiological data are available (e.g., EEG) without anatomical or connectivity data. In these cases, TVB platform includes normalized anatomical data (a parcellated cortical surface based on the MNI atlas) and a theoretical structural connectome based on the CocoMac database (3, 57). For stroke cases, while it is preferable to have anatomical data, it is still possible to run accurate simulations by manually modifying this provided structural connectome to exemplify the individual lesions.

### The Resulting TVB Models are Individualized

There is large consensus on the importance of individualized medicine as one of the means to improve medical care. In this sense, a central feature of TVB is its direct focus on individual subjects’ brain dynamics. The structural connectivity matrix of each individual drives the modeling producing the individualized simulated brain activity, whereas the applicability of previous studies has been at the group level (15). By generating reliable simulations, the system provides a window into the state of biophysical parameters associated with it in each person and hence enables the development of customized, individualized therapies and treatments.

There are a myriad of stroke therapies currently under investigation, including constraint-induced motor therapy (58–60), action observation therapy (61, 62), neurostimulation (e.g., transcranial magnetic stimulation and transcranial direct-current stimulation) (63, 64), robotic therapy (65, 66), and cellular-based (e.g., stem cell) therapies (67), that have shown limited degrees of effectiveness, due perhaps to the fact that they are not specifically targeting brain mechanisms responsible for individual dysfunction. This is a reflection of the paucity in our understanding of basic mechanisms generating individual brain dynamics. Having new hypotheses applicable to each patient will enable us to generate new therapeutic interventions that specifically target the elements producing particular brain states. Furthermore, the more we learn about basic processes based on animal studies for instance, the more we can modify current TVB local models and hence, obtain more sophisticated simulations.

### TVB Parameters can be Related to Other Network Metrics

An additional feature of parameters derived from TVB is that they can be contrasted with other measures. Our results showed a trend toward decreased global efficiency in stroke that measures the network’s capacity for communication, with greater efficiency indicating better overall communication (20, 49). In other words, network communication is impaired after stroke. Interestingly, degree centrality and betweeness centrality after stroke were not different from healthy controls probably due to the large variance of stroke size.

The negative correlation between global efficiency and the modeled long-range coupling provides unique insight into the network structure of the brain following stroke. We have previously observed increased long-range coupling after stroke, intuitively indicating a higher influence of local dynamics on brain activity than long-range dynamics. In this context, it is important to remember that the global model is derived from the structural connections between nodes, and hence, one would expect that shorter (direct) paths that originate from damaged nodes should be compromised. The graph analysis results suggest that the poststroke connectivity between nodes is done through less efficient, longer paths (20). Therefore, decreased global efficiency and increased long-range coupling after stroke suggest a breakdown in the ability to transfer information between regions, weighting the activity toward local dynamics. Our findings thus highlight the global impact of stroke, despite its relatively focal damage. This novel finding in stroke is consistent with studies in other neurological diseases, such as schizophrenia, where imbalances between local and global dynamics, specifically a breakdown of local structure and a shift toward global dynamics have been suggested (68).

### Limitations

The Virtual Brain as any modeling approach is laden with limitations. Among them:

1. The fact that TVB simulations depend on structural connectivity assumes the structural matrices having reasonable reliability. This is very relevant in stroke because the damage can produce mechanical distortions of tissue. In our case, we have used TVB transplant to minimize these issues. Additionally, there are many definitions of “weights” of connections (69, 70) although novel approaches promise at least high intraindividual reliability in the reconstruction (71). In our case, we used a surrogate measure reflecting the “number of fibers per pathway.” This is the reason why we normalized the number of streamlines between nodes by the FA of the particular pathway.
2. The weights of connections are currently based on the size (number of streamlines) of the pathways, yet the particular features of the synaptic connections are not taken into consideration. For example, the penetrance of a smaller pathway could be larger than a bigger pathway if the former establishes the synaptic contact with more proximal versus distal dendrites. This type of information is available for other species but is not yet known in humans.

### Future Directions and Clinical Impact

The ability of generating a virtual brain from any individual opens up an interesting venue for therapeutics. Once a hypothesis is derived from the biophysical parameters affected by the stroke, the effects on brain dynamics can be tested within the TVB platform by modifying the parameters for an individual case. In this way, TVB can be used as a test for potential therapeutic interventions before they are tested in animal models or individual patients.

The Virtual Brain thus has the potential to revolutionize stroke treatment in the future, by allowing for:

1. The application to “big data.” While the current study used a smaller sample size, once we have parameter changes, future studies can more readily utilize TVB in a large number of patients.
2. The ability to study longitudinal brain changes in stroke, from acute and sub-acute to chronic stroke. Because of the predictive potential of TVB, the inclusion of patients at early stages can provide the identification of powerful biomarkers for recovery.
3. The individualization of treatment with minimal input: one single MRI scan including the anatomical scan, DTI, and resting state fMRI.
4. The ability to perform whole-brain modeling, integrating the particular intercommunication between nodes (DTI derived) to local biophysical models associated with concrete basic functional parameters.
5. The opportunity to identify tangible targets for treatment that are testable within the application itself.
6. An open source platform: it is possible to add new, more sophisticated mesoscopic and microscopic models via the open source nature of TVB. Therefore, new developments on basic physiological knowledge can be easily integrated in the future.
7. Allowing the simulation of resting state brain activity, as was done in this study, but also of evoked responses through a built-in feature that allows for the stimulation of brain areas, with features determined by the modeler.

## Author Contributions

All authors had full access to all data in the study and take responsibility for the integrity of the data and the accuracy of the data analysis. Study concept and design: AS, VJ, and MF. Analysis and interpretation of data: AS, MF, JR, VJ, EC, ADS, and AM. Drafting of the manuscript: MF, AS, VJ, and ADS. Critical revision of the manuscript for important intellectual content: AS, VJ, JR, and AM Statistical analysis: EC. Obtained funding: AS, VJ, and AM. Study supervision: AS and VJ.

## Conflict of Interest Statement

The authors declare that the research was conducted in the absence of any commercial or financial relationships that could be construed as a potential conflict of interest.
